# Supplementary material for: Reassortment Network of Influenza A Virus
Source: Front Microbiol. 2021 Dec 16;12:793500. doi: 10.3389/fmicb.2021.793500 (PMC8716808; doi:10.3389/fmicb.2021.793500)

Supplementary Figure SF1. The detailed type result for PB2 segment. The year range, hosts, locations and subtypes are shown after each PB2 type, where the circles represent hosts and the rectangles represent locations. The hosts and locations are distinguished by different colors.

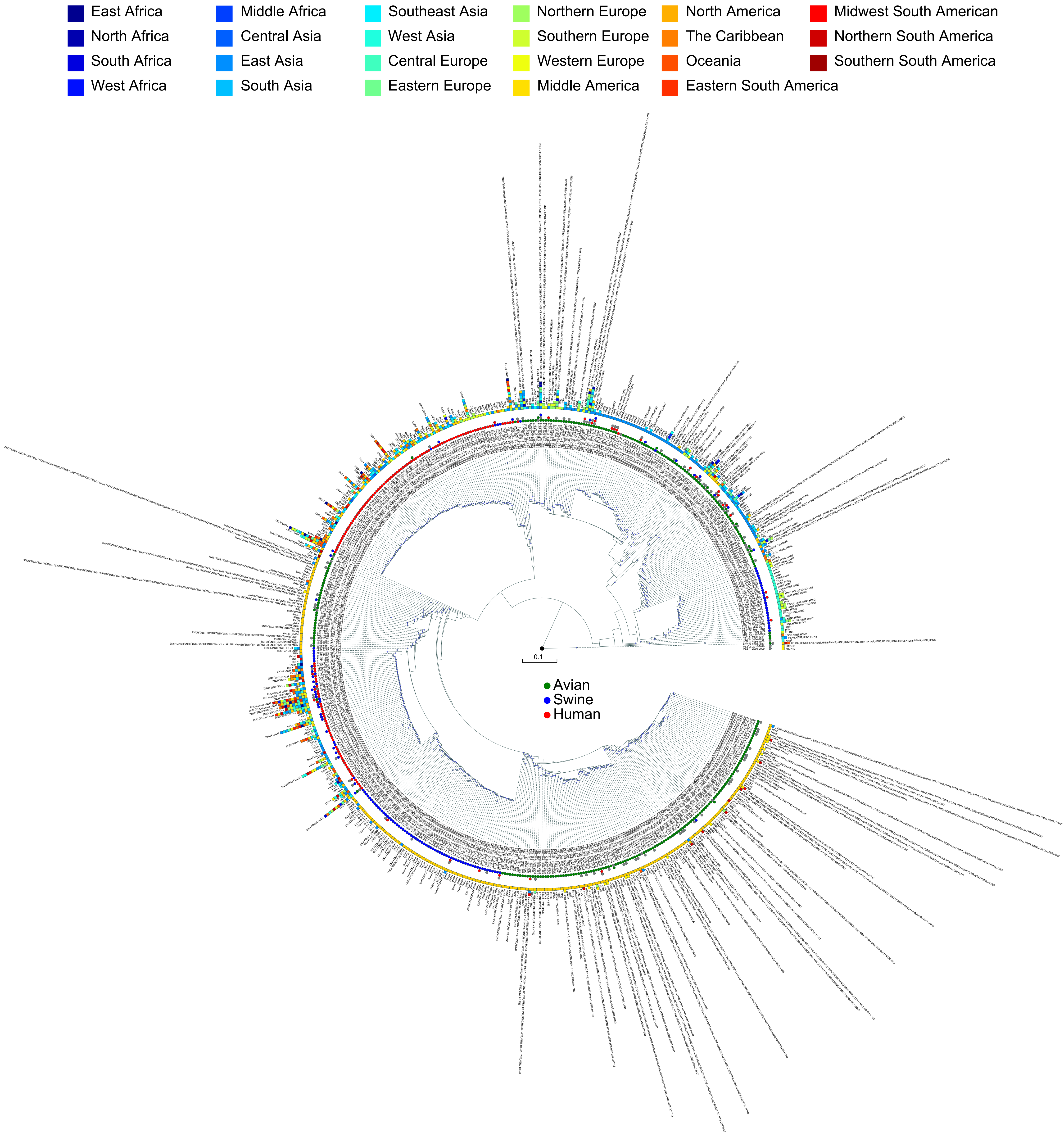

Supplement: Supplementary file 2 [file Data_Sheet_2.ZIP › Supplementary Figure SF1.pdf]
