# Supplementary material for: Reassortment Network of Influenza A Virus
Source: Front Microbiol. 2021 Dec 16;12:793500. doi: 10.3389/fmicb.2021.793500 (PMC8716808; doi:10.3389/fmicb.2021.793500)

Supplementary Figure SF10. The gene flow network of IAVs (with location information annotated). Each node is a genotype of IAVs and the edge represents gene segment flow by reassortment between two genotypes. The locations are distinguished by different colors.

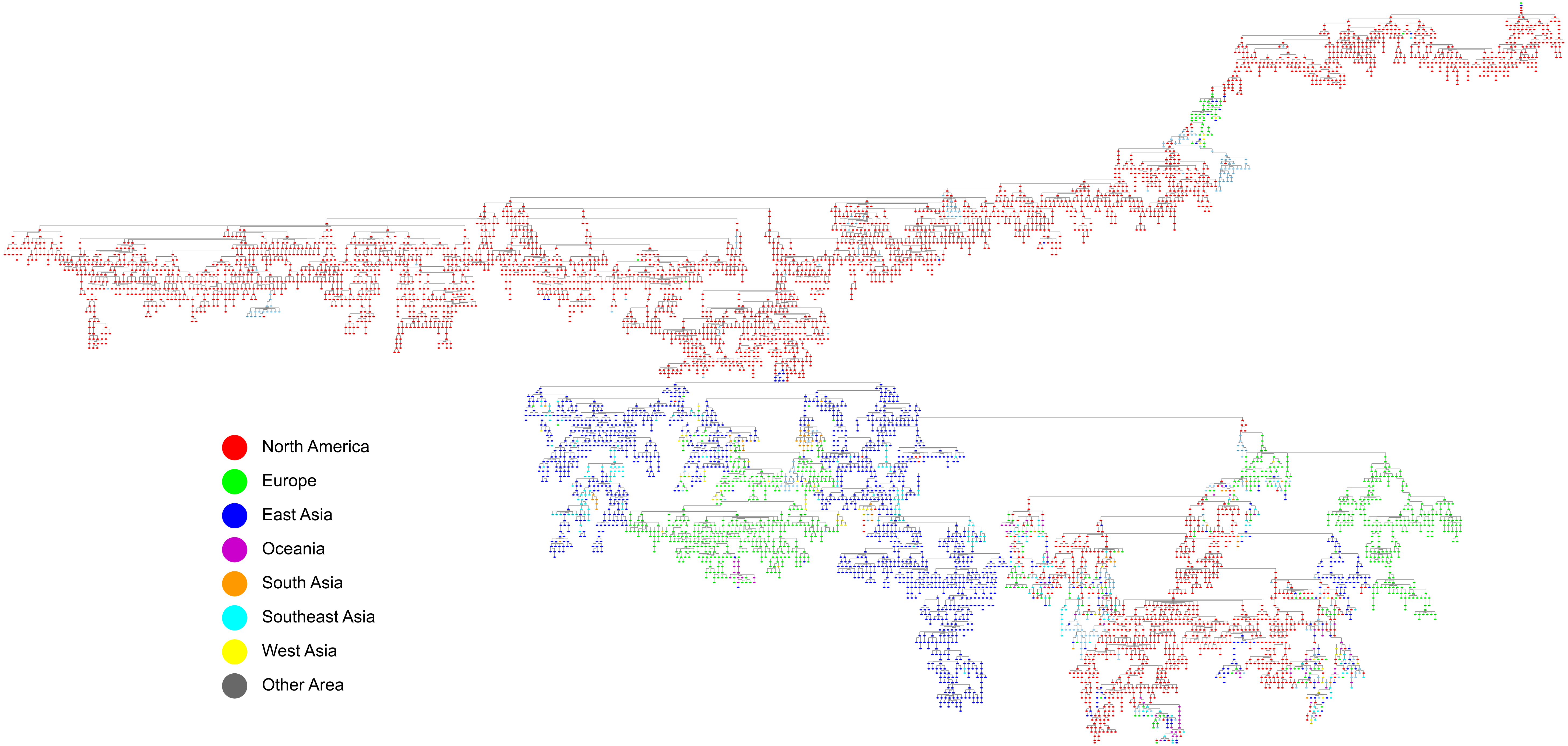

Supplement: Supplementary file 2 [file Data_Sheet_2.ZIP › Supplementary Figure SF10.pdf]
