# Supplementary material for: Reassortment Network of Influenza A Virus
Source: Front Microbiol. 2021 Dec 16;12:793500. doi: 10.3389/fmicb.2021.793500 (PMC8716808; doi:10.3389/fmicb.2021.793500)

Supplementary Figure SF3. The detailed type result for HA segment. The year range, hosts, locations and subtypes are shown after each HA type, where the circles represent hosts and the rectangles represent locations. The hosts and locations are distinguished by different colors.

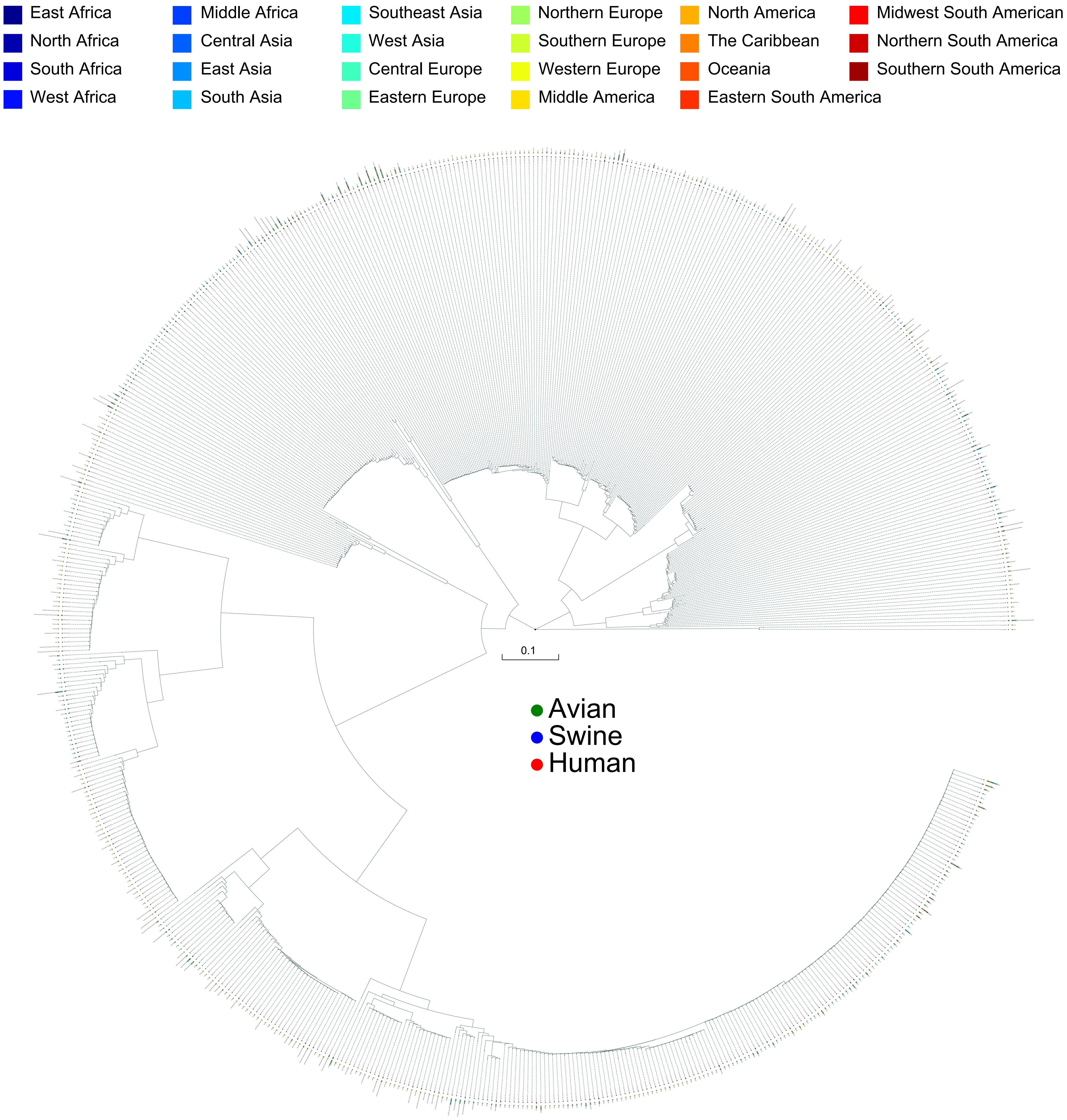

Supplement: Supplementary file 2 [file Data_Sheet_2.ZIP › Supplementary Figure SF3.pdf]
