# Supplementary material for: Reassortment Network of Influenza A Virus
Source: Front Microbiol. 2021 Dec 16;12:793500. doi: 10.3389/fmicb.2021.793500 (PMC8716808; doi:10.3389/fmicb.2021.793500)

Supplementary Figure SF4. The detailed type result for NP segment. The year range, hosts, locations and subtypes are shown after each NP type, where the circles represent hosts and the rectangles represent locations. The hosts and locations are distinguished by different colors.

East Africa	Middle Africa	Southeast Asia	Northern Europe	North America	Midwest South American
North Africa	Central Asia	West Asia	Southern Europe	The Caribbean	Northern South America
South Africa	East Asia	Central Europe	Western Europe	Oceania	Southern South America
West Africa	South Asia	Eastern Europe	Middle America	Eastern South America	

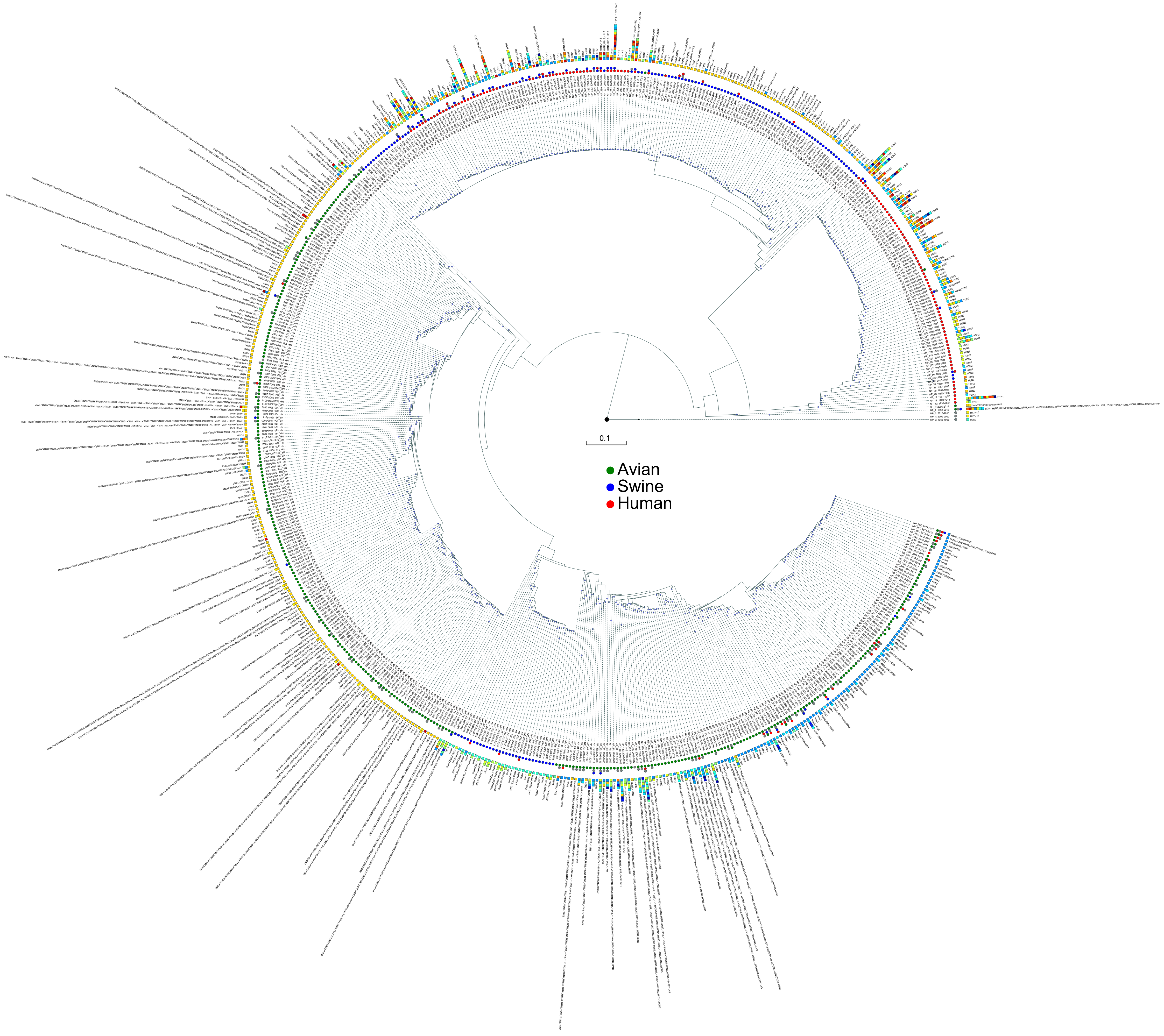

Supplement: Supplementary file 2 [file Data_Sheet_2.ZIP › Supplementary Figure SF4.pdf]
