# Supplementary material for: Reassortment Network of Influenza A Virus
Source: Front Microbiol. 2021 Dec 16;12:793500. doi: 10.3389/fmicb.2021.793500 (PMC8716808; doi:10.3389/fmicb.2021.793500)

Supplementary Figure SF8. The reassortment history of IAVs. Each sector represents a location, while the polar axis represents the years. Each circle represents a virus, with different colors to indicate the hosts. The line from source to target represents the parental virus produces the reassortant virus. Intra-Locations and Inter-Locations reassortment are indicated by green and red lines, respectively.

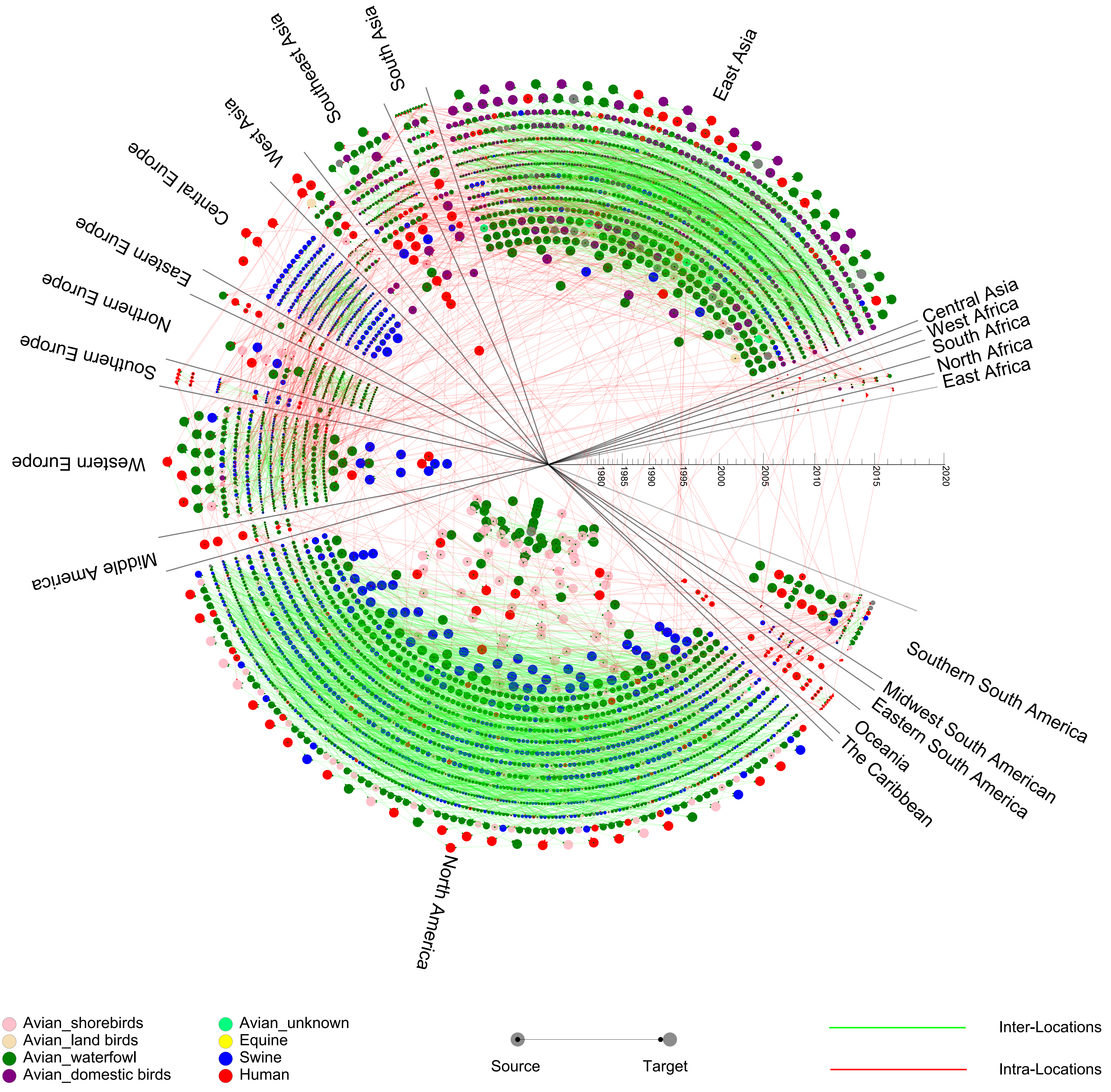

Supplement: Supplementary file 2 [file Data_Sheet_2.ZIP › Supplementary Figure SF8.pdf]
